# Lineages of the Cardiac Conduction System

**DOI:** 10.3390/jcdd4020005

**Published:** 2017-05-02

**Authors:** Rajiv Mohan, Bas J. Boukens, Vincent M. Christoffels

**Affiliations:** Department of Medical Biology, University of Amsterdam, Amsterdam, 1105 AZ, The Netherlands; r.a.mohan@amc.uva.nl (R.M.); b.j.d.boukens@amc.uva.nl (B.J.B.)

**Keywords:** cell lineage, lineage tracing, cardiac conduction system, cardiac conduction system lineage, genetic fate map, genetic inducible fate map, cardiac development

## Abstract

The cardiac conduction system (CCS) initiates and coordinately propagates the electrical impulse to orchestrate the heartbeat. It consists of a set of interconnected components with shared properties. A better understanding of the origin and specification of CCS lineages has allowed us to better comprehend the etiology of CCS disease and has provided leads for development of therapies. A variety of technologies and approaches have been used to investigate CCS lineages, which will be summarized in this review. The findings imply that there is not a single CCS lineage. In contrast, early cell fate decisions segregate the lineages of the CCS components while they remain connected to each other.

## 1. Introduction

The heart is a pump that generates pressure to maintain blood circulation. The cardiac conduction system (CCS) coordinates the rhythmic contractions of the atria and ventricles by a tight control of electrical impulse propagation. Failure of the correct patterning and formation of the CCS components leads to its dysfunction, resulting in arrhythmias [1]. Therefore, it is important to gain insight into these developmental processes and the cells involved.

Many cell fate decisions are made while an omnipotent zygote, by cell divisions, grows into a mature multicellular organism, consisting of hundreds of different specialized cell types. The developmental history (or ancestry) of a specialized cell type is referred to as a lineage and can theoretically be traced back to the zygote. Usually, it is traced back to a progenitor cell type in which a lineage decision was made. Multiple lineages can derive from such a decision point. Identifying the lineages of the CCS will enable us to determine the consecutive cell fate decisions made during development and provide insight into the underlying molecular mechanisms.

The CCS’s lineages have been investigated using a variety of approaches, sometimes leading to contradictory interpretations. First, we briefly summarize the composition and function of the components of the CCS and the common ancestry of the cells comprising them. Then, techniques used to trace the CCS’s lineages and their limitations will be reviewed. Finally, we summarize the available data and present a model for the CCS’s lineages. Our literature research indicates that the CCS forms from multiple lineages that segregate early in embryonic development. These early lineage segregations of the progenitor populations result in sharp boundaries between the CCS components to which a progenitor population contributes.

## 2. The Cardiac Conduction System

**Figure 1:**
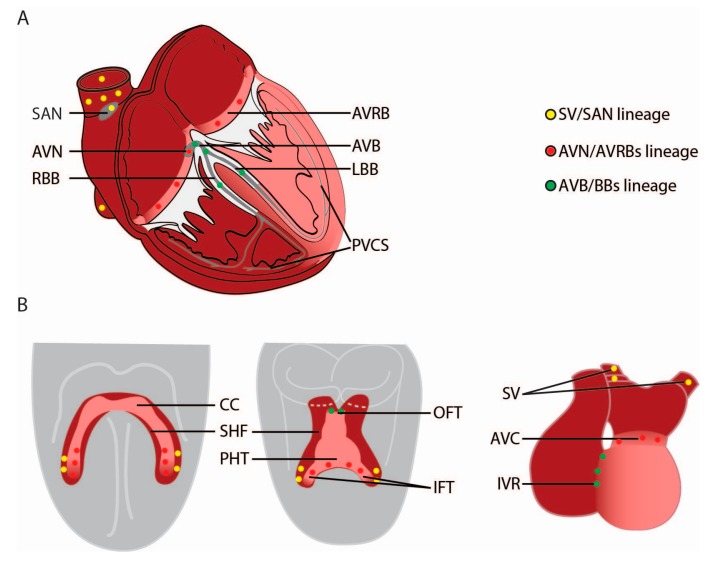
Developmental origin of the cardiac conduction system components. (**A**) The mature cardiac conduction system (CCS, grey) consists of the sinoatrial node (SAN), the atrioventricular node (AVN) and ring bundles (AVRBs), the atrioventricular bundle (AVB), left and right bundle branches (LBB and RBB, respectively) and the peripheral ventricular conduction system (PVCS). The contributions of the first and second heart field (FHF and SHF, respectively) to the adult myocardium are visualized in pink (FHF-derived) and dark red (SHF-derived); (**B**) Cardiogenic mesodermal cells of the FHF differentiate and form the cardiac crescent (CC) at E7.5. The primary heart tube (PHT) forms through fusion of the cardiac crescent (E8) and subsequently forms the myocardium of the left ventricle and part of the atrioventricular canal (AVC). Cardiogenic mesodermal cells of the SHF are continuously added to the arterial and venous pole of the heart tube and form the other myocardial components of the heart. The locations of the progenitor cells of the CCS components are depicted by yellow circles for the SV/SAN, red circles for AVN/AVRBs and green circles for AVB/BBs. Abbreviations: IFT, inflow tract; IVR, interventricular ring, OFT, outflow tract; SV, sinus venosus.

The adult CCS initiates and propagates the electrical impulse, thereby activating the atria and subsequently the ventricles in a regular rhythm (Figure 1A). The electrical impulse is generated in the sinoatrial node (SAN), the dominant pacemaker of the heart, positioned at the border of the superior caval vein and the right atrium. The electrical impulse travels from the SAN through the atria to reach the atrioventricular node (AVN) and the atrioventricular ring bundles (AVRBs). The AVN is located dorsally at the base of the interatrial septum and propagates the impulse slowly. After being delayed in the AVN, the electrical impulse is rapidly conducted through the atrioventricular bundle (AVB), which divides into the left and right bundle branches (LBB and RBB) in the interventricular septum (IVS). The electrical impulse continues into the left and right peripheral ventricular conduction system (PVCS) or Purkinje fibers, which is an intricate cellular network that lies just below the endocardium. From here, the ventricular working cardiomyocytes (WM) are initiated to depolarize and contract, resulting in blood pumping into the aorta and pulmonary artery [2].

Although CCS components share important features, they also differ markedly in several aspects, suggesting that there are multiple distinguishable CCS lineages. These different properties include spontaneous depolarization rate, degree of intercellular coupling and action potential morphology. For example, the SAN is the dominant pacemaker because it has the highest spontaneous depolarization rate among the CCS components. In addition, the SAN and AVN conduct the impulse slowly, whereas the ventricular conduction system (VCS), consisting of the AVB, BBs and PVCS, conduct it rapidly [3,4,5]. These particular features of CCS components are the result of lineage decisions made during the differentiation of CCS progenitor cells, which are important for their proper function.

The structure and location of CCS components may differ between species. In mammals, the AVB is located at the crest of the IVS, whereas in birds it is buried within the IVS [6,7]. In birds, inductive cues from the endothelium of coronary arteries play a role in the formation of a peri-arterial PVCS [7,8]. These inductive cues are less important in mouse PVCS development [9,10]. Nevertheless, the structure, function and relative locations of the CCS components are in general very similar between species, indicating that the lineage contributions to the different CCS components are conserved.

## 3. Techniques Used in CCS Lineage Tracing

Over the years, a variety of techniques have been developed for interrogating cell lineages [11,12,13,14]. The common goal of these techniques is to label a particular cell or cell population at a certain developmental stage and to analyze the descendants at a later developmental stage to assess the fate of the initially labeled cells. These labeling techniques are usually combined with phenotypic analysis (e.g., gene expression) of both the labeled cells and their descendants to establish lineage relationships. Each technique has its own strengths and limitations. In this section, we limit ourselves to the discussion of cell lineage tracing techniques that have been used to assess the CCS’s lineages.

### 3.1. Labeling Cells with Dyes

Present dye-mediated labeling techniques use a fluorescent dye that is injected into a cell or tissue which incorporates into the cell membrane. The cell identity of the labeled descendants is analyzed at a later developmental stage. In chickens, this approach can be performed in ovo [15]. In mice, however, dye labeling occurs ex utero to gain physical access to the embryo, which immediately represents the biggest limitation. In the absence of a maternal nutrition supply the process of development arrests which makes these experiments only suitable for short term fate maps (24–48 h). Other limitations are dilution of the dye after multiple rounds of cell division and uncertainty of which cells or cell types have been labeled in a tissue. This type of lineage tracing was important in establishing the fate of the posterior part of the cardiogenic mesoderm. Cells located more cranially within the posterior part contribute to the atrioventricular canal (AVC) and atria, whereas cells located more caudally contribute to the sinus venosus (SV) [16].

### 3.2. Viral Transduction of a Reporter Transgene

Viral transduction using a replication defective virus delivers a reporter transgene (i.e., genetic marker functioning as a reporter) to label a cell or tissue [17]. The transgene is driven by a ubiquitously active promoter and expresses a reporter gene (encoding an easily detectable protein). The expression of the reporter transgene can be analyzed at a later developmental stage in the progeny of the initially labeled cells. Transduction of cells is mainly performed by adenoviruses or retroviruses [18]. An adenovirus introduces the reporter transgene as a double stranded episomal DNA molecule into the cell. The episomal DNA molecules are diluted after several rounds of cell division. Retroviruses introduce a reporter transgene that integrates at a random position into the host genome. The reporter transgene is passed on to all daughter cells giving a major advantage over adenovirus and dye labeling methods. A limitation, however, is different transduction efficiencies between cells. For example, retroviruses only label dividing cells. Another limitation of retroviruses is that the randomly integrated reporter transgene can be silenced in a subpopulation of daughter cells, generating an incomplete fate map [13]. Moreover, in mammals, the period between labeling and fate analysis is short because the virus is applied ex utero. Adenoviral and retroviral lineage tracing studies led to the discovery that the VCS and surrounding ventricular WM share a common myocardial progenitor population present in the looped heart tube [19].

### 3.3. Genetic Fate Mapping—Cre-LoxP System

**Figure 2:**
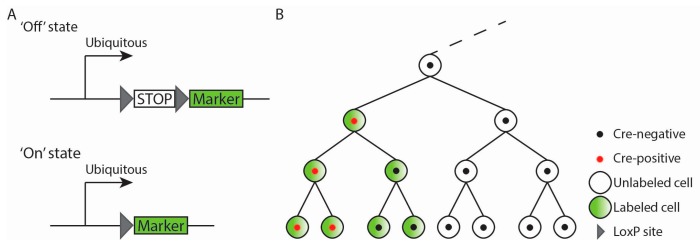
Genetic fate mapping relies on recombinase-mediated excision to switch ‘On’ and label cells and their progeny. (**A**) The ubiquitously active reporter transgene consists of a STOP-gene flanked by two LoxP sites (‘floxed’ STOP-gene) and a gene encoding a marker. Cre recombinase recognizes the two LoxP sites and excises the STOP cassette. The reporter is in the ‘Off’ state, but after Cre-mediated excision switches to the ‘On’ state, expressing the marker and thereby labels the cell; (**B**) Schematic representation of genetic fate mapping. Only cells that express Cre during development will switch the reporter transgene to the ‘On’ state. As this is a heritable recombination event, all the descendants of these cells will be labeled likewise, independent of Cre expression, and can be characterized.

To permanently label a chosen cell population and its descendants, researchers have used cell- or tissue-specifically expressed recombinase that irreversibly activates a reporter transgene. The widely used site-specific recombinases Cre and Flp recognize LoxP or FRT sites, respectively, in the genome and excise or invert the region in between [20,21]. In mice, genetic fate mapping is usually performed using the Cre-LoxP system. Most commonly, cell- or tissue-specific (surrogate for lineage-specific) Cre expression is achieved by driving the *Cre* gene under control of a tissue-specific gene such that the recombinase recapitulates the spatio-temporal expression pattern of that particular gene. The recombinase-activated reporter is an independent transgenic construct that consists of a ubiquitously active promoter driving a cassette with transcriptional termination and poly-adenylation sites flanked by two LoxP sites (a “floxed” STOP-gene) and a gene encoding for a protein, usually a fluorescent protein functioning as a marker (Figure 2A). The reporter can switch from an ‘Off’ state due to the floxed STOP-gene into the ‘On’ state, expressing the fluorescent protein, thereby labeling the cell. Alternatively, a multicolor reporter can be used. For example, in the mTmG reporter, prior to Cre-mediated excision, the floxed stop-gene encoding tdTomato is expressed, after Cre-mediated excision the gene encoding GFP [22]. Only in *Cre*^+^ cells, the floxed gene can be excised and the marker expressed. As this is a heritable recombination event, marker expression no longer depends on Cre activity, and all the descendants of the labeled cell will be labeled and can be characterized (Figure 2B).

The major advantages of this method are (1) labeling is non-invasive, i.e., no physical access to the embryo is required, (2) long-term cell lineage tracing is possible, even into adulthood, and (3) no label dilution occurs. However, this method has major potential pitfalls as well. Recombination of the LoxP sites occurs stochastically and depends on the concentration and time that Cre is present and on the genomic integration site of the floxed reporter transgene. Consequently, recombination may not occur in a *Cre*^+^ cell when the Cre concentration is low or when Cre is expressed for a brief period of time. In addition, the type of floxed reporter transgene used influences the resulting fate map. An example of an incomplete fate map is the first lineage analysis conducted of the *Isl1*^+^ cells in the embryo. It was concluded that *Isl1*^+^ mesodermal cells contribute to the right ventricle (RV), outflow tract (OFT) and atria with only a minor contribution to the left ventricle (LV) [23]. Later, two other *Isl1*^+^ fate maps were generated with different outcomes. One used *Isl1*-driven Cre and a *Gata4* locus-based reporter and the other used an *Isl1*-driven inducible Cre (see Section 3.4. for a detailed explanation) and *R26R-lacZ* reporter [24,25]. Both studies observed a broader labeled domain including (a major part of) the LV, indicating that the LV progenitors also express *Isl1*, but more briefly. A second major pitfall is low Cre expression (undetectable for the observer), which nonetheless results in labeling. This may result in erroneous interpretations regarding the origin of a lineage. One example is the conclusion that (IVS) myocytes are derived from *Tbx18*^+^ epicardial cells [26], while in fact, these myocytes express low levels of *Tbx18* (or *Cre*) themselves [27]. Another limitation of genetic fate maps arises when the labeled tissue at the moment of fate analysis still expresses Cre. In this situation, the labeling could have taken place at any stage during development, and the link between Cre-expressing progenitors and the labeled cell population at the stage of fate analysis cannot be made. However, under such circumstances, unlabeled tissues can still be very informative because they most likely do not originate from *Cre*^+^ progenitors. In addition, if the recombinase during development is expressed by multiple cell populations at a particular stage of development or by several cell populations at multiple developmental stages, it is not possible to identify the progenitors of the labeled tissue [14]. An additional caveat is inactivation of the gene in which *Cre* is inserted. The resulting haploinsufficiency could influence the contribution of the *Cre*^+^ cell population to a tissue, thereby affecting the generated fate map. Finally, it has been reported that the presence of Cre or the inducible Cre in a cell can result in apoptosis and cell cycle arrest, thereby affecting development and the resulting fate map [28].

### 3.4. Genetic Inducible Fate Mapping—Inducible Cre-LoxP System

Temporal control of recombinase activity solves two major shortcomings of the above-described Cre-mediated lineage analysis. Firstly, temporal control can discriminate between cell populations expressing Cre at multiple development stages. Secondly, temporal control overcomes the problem of Cre still being expressed in the labelled tissue at the moment of analysis. The recombinase is modified in such a way that it is only able to recombine and mark cells in the presence of a ligand which can be introduced at any developmental stage [29,30,31]. In case of the widely used inducible CreER^T2^ or MerCreMer, the recombinase is fused to a mutated estrogen receptor ligand binding domain (ER-LBD) which sequesters CreER^T2^ into the cytoplasm by binding to a cytoplasmic protein complex [32,33]. Administered ligand tamoxifen (*in vivo* converted to the active form 4-hydroxytamoxifen) or 4-hydroxytamoxifen binds to the ER-LBD, which causes a conformational change releasing CreER^T2^ from the cytoplasmic protein complex. Subsequently, CreER^T2^ translocates to the nucleus where it recombines a reporter thereby labeling the cell. Shortcomings of this technology are incomplete labeling of the *Cre*^+^ population due to inefficient recombination, ligand-mediated embryonic toxicity and premature labor, and ‘leakage labeling’, due to translocation of the inducible Cre to the nucleus in the absence of the ligand [14].

The hyperpolarization-activated cyclic nucleotide-gated channel (Hcn4) is an ion channel responsible for the funny current (*I*_f_), important for the pacemaker potential. The gene is dynamically expressed in most cardiac progenitor populations and cardiomyocytes during development before it becomes confined to the SAN, and finally the CCS [2,34,35]. A genetic fate map using constitutively active Cre to assess the contributions of *Hcn4*^+^ cells would yield a broadly labeled heart. Using an *Hcn4-CreER^T2^*, *Hcn4*^+^ populations at particular developmental stages were labeled. Labeling of the *Hcn4*^+^ cardiogenic mesoderm/primary heart tube confirmed that these cells only contribute to the LV [35].

### 3.5. Retrospective Clonal Analysis—nLaacZ Reporter

**Figure 3:**
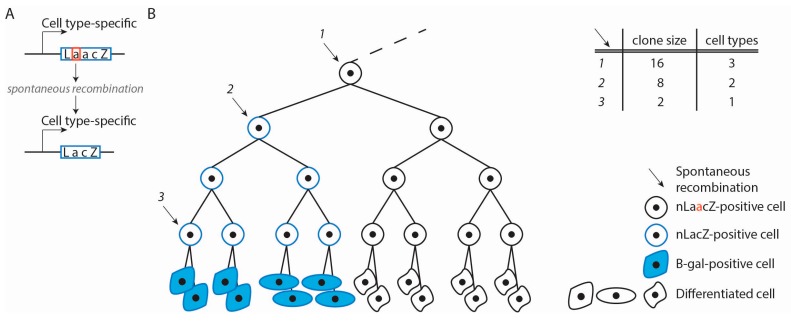
Retrospective clonal analysis using the transgene *nLaacZ*. (**A**) The *nLaacZ* transgene harbors an intergenic duplication resulting in a truncated coding sequence. A rare spontaneous recombination event removes the duplication, thereby generating a functional *LacZ* gene encoding nuclear localized β-galactosidase; (**B**) Schematic representation of *nLaacZ*-based retrospective clonal analysis visualizing one recombination event (2). Spontaneous recombination of the *nLaacZ* transgene can occur in any cell and at any time during development and labels a cell and its descendants. The descendants form a clone because they originate from the same cell. Clone size is proportional to the time between recombination and analysis. Labeling of a more undifferentiated cell early in development (1) results in a bigger clone consisting of several cell types compared to labeling at a later developmental stage in a more fate restricted cell (2 and 3). A temporal history of a lineage is reconstructed by characterizing multiple clones that differ in size, distribution and cell type.

In retrospective clonal analysis of the CCS’s lineages, the transgene *nLaacZ* was used. The *nLaacZ* transgene harbors an intragenic duplication, resulting in a truncated coding sequence. Labeling of a cell and all its descendants depends on a rare recombination event that occurs randomly, removing the duplication and thereby generates a functional *nLacZ* gene coding for nuclear-localized β-galactosidase (Figure 3A) [12,36]. It is retrospective because the fate of the initially labeled progenitor is observed, but the developmental stage and location of the labeled progenitor is not known and has to be retrospectively deduced. A typical library consists of hundreds of embryos, the majority of which are without labeled cells, and some embryos with a clone that differs in size, distribution and cell type. Using a statistical test, it is determined whether or not the labeled cells are clonally related (originated from a single labeled progenitor). It relies on the premise that clone size (cell number) correlates with the time between recombination and fate analysis, i.e., the number of cell divisions that could have taken place. Moreover, using this approach, dispersed growth or cell movement can be discovered because these processes lead to single cell-derived descendants that are broadly distributed rather than confined. By characterizing multiple clones that differ in size, distribution and cell type and by following these premises, a temporal history of a lineage is reconstructed (Figure 3B).

The technique is non-invasive and the labeling is permanent, which allows for long-term analysis. More importantly, every cell in the embryo can be labeled and its descendants can be characterized. Therefore, it is not necessary to have a preconceived idea about the possible progenitor population of a tissue followed by generating a dedicated transgenic *Cre* allele. A disadvantage of spatio-temporal random labeling of cells is that the exact developmental stage and location of the initially labeled progenitor are unknown. Moreover, differences in the proliferation rate of daughter cells are not taken into account. If the daughter cells form subpopulations with different proliferation rates, it becomes especially difficult to establish an order in the temporal history of a lineage. Despite these disadvantages, this approach has proven to be very useful in reconstructing lineages and the order of cell fate decisions. This approach was used in mice and confirmed the observation made in chickens that VCS and ventricular WM cardiomyocytes originate from a common progenitor population [37].

## 4. General Description of the Origin of the Heart Components

Just after gastrulation, progenitor cells of the heart form two epithelial cell populations in the cranio-lateral mesoderm, known as the cardiogenic mesoderm or first heart field (FHF). Subsequently, the cranial parts fuse at the midline and the cardiac crescent is formed. Folding of the embryo, foregut formation and fusion of the cardiac crescent in a cranial to caudal fashion results in the formation of the primary heart tube. Micro particle labeling of chicken embryos revealed that the initial primary heart tube gives rise to only the LV and part of the AVC [38]. This was also shown to be true for mammals. The first-formed cardiomyocytes express *Hcn4*, and labeling of the *Hcn4*^+^ cells using *Hcn4-CreER^T2^* and a reporter revealed that the cardiac crescent forms the LV and part of the AVC in mouse development [35]. At this stage, cardiomyocytes in the primary heart tube do not proliferate [39]. Dye labeling, transgenic labeling using the long half-life of β-galactosidase, viral transduction, in vivo ablation and *Isl1*-Cre mediated lineage tracing experiments showed that the surrounding cardiogenic mesoderm contributes to both the venous and atrial pole of the heart tube and gives rise to the other components of the definitive heart [23,39,40,41,42]. This progenitor pool is referred to as the second heart field (SHF). The inflow tract (IFT) of the primary heart tube becomes the major part of the AVC, while the newly added IFT cells go on to become the atrium. Finally, the cells of the definitive SV are added. At the arterial pole, the OFT of the primary heart tube becomes embryonic RV, newly added cells from the SHF form the fetal OFT and subsequently the RV outflow tract. The cells added last form the intrapericardial portion of the aorta and pulmonary trunk. Taken together, the FHF forms the initial primary heart tube which only forms the definitive LV and part of the AVC. The cardiogenic mesoderm of the SHF in the dorsal pericardial wall forms the remaining structures.

In the forming heart, the SAN develops in the SV bordering the RA. Theoretically, this means that it is formed from the latest added SV cells at the venous pole. The AVN is formed within the AVC, and as a consequence is likely to be derived from the FHF-derived IFT of the primary heart tube. The AVB and BBs are located on the crest of the IVS, in between the LV and RV. Logic dictates that the AVB is derived from the progenitors at the border between the FHF (LV) and SHF (RV), the LBB from FHF, and RBB from SHF progenitors. The PVCS in the LV is formed from cells originating from the primary heart tube derived from the FHF. The PVCS in the RV is formed within the RV compartment that is added to the heart at the arterial pole after the primary heart tube has been formed (Figure 1B).

## 5. The Sinoatrial Node is Formed by Early Segregated Mesodermal Cells

The definitive SV consists of the SAN, the left, common and right sinus horns and the venous side of the venous valves. The SAN primordium develops within the SV, directly adjacent to the embryonic right atrial wall, and can be discriminated morphologically around E10 of mouse development [43]. The SV/SAN selectively express *Tbx18* but not *Nkx2-5* [44,45]. Lineage analysis using *Tbx18*-driven Cre and *Nkx2-5*-driven Cre confirmed that the SV and SAN are derived from *Tbx18*^+^ *Nkx2-5*^−^ precursors, and that a sharp lineage boundary is present between the SV/SAN and the *Tbx18*^−^ *Nkx2-5*^+^ *Nppa*^+^ atrial myocardium [44,45,46]. Indeed, when embryonic atrial cardiomyocytes were labeled from E10.5 onwards using an *Nppa*-driven Cre, no contribution was observed to the SV and SAN, indicating that SAN cardiomyocytes do not originate from atrial cardiomyocytes by recruitment, but from the *Tbx18*-expressing SV progenitors [47]. Moreover, cultured embryonic explants of *Tbx18*^+^ mesodermal cells differentiated into *Nkx2-5*^−^ cardiomyocytes with a higher beating frequency than explants from embryonic ventricular cardiomyocytes [45,48]. Dye labeling of the *Tbx18*^+^ pericardial wall further indicated that the *Tbx18*^+^ mesoderm probably encompasses the SV progenitor population [48]. *Shox2* becomes expressed in the SV at E9.5, and like *Tbx18* remains expressed in the mature SAN [49]. *Shox2*-driven Cre specifically labeled the SV and SAN. Both Tbx18 and Shox2 are important for the formation of the SAN and seem to function as specifiers of the SV/SAN lineage [45,50].

The contribution of the posterior part of the SHF at the 4 and 6-somite stage was examined using dye labeling of cultured mouse embryos [16]. Posterior SHF cells located more cranially contributed to the AVC and atria, whereas cells more caudally located contributed to the SV. In addition, a study on chickens using dye labeling and focusing on the origin of the SAN revealed that the SAN progenitors in the early embryo are located in a region posterior of the *Nkx2-5*^+^ and *Isl1*^+^ cardiogenic mesoderm. This is in line with the position of the *Tbx18*^+^ *Nkx2-5*^−^ progenitor mesoderm in the mouse. Furthermore, specification of the SAN progenitors was indicated to occur when they are still part of the cardiogenic mesoderm [51].

Using *Nkx2-5-Ires-Cre*, it was shown that the SV and SAN are derived from Nkx2-5^−^ progenitors [46,52]. More recently, an *Nkx2-5*^+^ lineage tracing was performed and the percentage of labeled cells in CCS components was assessed. The SAN, in contrast to the other components, showed a low labeling percentage [35]. Although not unambiguously, these fate map experiments suggest that SAN progenitors do express *Nkx2-5*. The low recombination efficiency suggests that *Nkx2-5* is either expressed at a low level or for a brief period in the SAN progenitors. A more careful analysis of the SV/SAN cardiac progenitors revealed that these cells briefly express both *Nkx2-5* and* Isl1* around E7.0. This expression is lost before the onset of *Tbx18* expression approximately two days before differentiation [48,53,54]. The brief period of *Nkx2-5* expression could explain the absence of SAN labeling in the *Nkx2-5-Ires-Cre* experiment [48,52]. In conclusion, Tbx18^+^ mesodermal cells form the SV and lose expression of *Nkx2-5* and *Isl1* before they initiate Tbx18 expression and subsequently differentiate into cardiomyocytes.

The *Isl1*^+^ progenitor population has been studied using *Isl1-Cre* and *Isl1-MerCreMer* alleles in combination with different reporters [23,24,25,35]. Concerning the SAN lineage, the first Cre-LoxP system-based *Isl1*^+^ lineage tracing did not specifically mention SAN labeling. However, the IFT of the E8.5 heart tube was shown to be labeled [23]. In another study, an *Isl1-Cre* fate map showed almost complete labeling of SAN cardiomyocytes [35]. However, *Isl1* remains expressed in the mature SAN [55,56]. Temporal control using an inducible Cre allows for labeling of only early *Isl1*^+^ mesodermal cells and solves the problem that occurs when Cre is still expressed in the labeled tissue at the moment of fate analysis. Tamoxifen induced labeling specifically at E6.0 using *Isl1*-*MerCreMer* resulted in labeling of the SV. Labeling at E9.0 could have resulted in sparse labeling of the SAN. However, no SAN marker was used to identify the SAN cardiomyocytes [25]. After the onset of *Tbx18* expression in the cardiogenic mesoderm, there are two expression domains present within the caudal SHF, a *Tbx18^+^* and an *Isl1*^+^ domain. The two expression domains mostly do not overlap except for one region, where one day later in development the embryonic SAN cardiomyocytes (*Tbx18*^+^, *Isl1*^+^, *Tbx3*^+^ and *Hcn4*^+^) can be discriminated [48]. Based on these observations, we conclude that the SAN is derived from *Isl1*^+^ progenitor cells.

In conclusion, the atria, sinus horns and SAN are formed by accretion of SHF progenitors at the venous pole. Yet, lineage analysis has revealed that the SV/SAN and atria originate from distinct progenitor pools, respectively, suggesting an early lineage segregation and cell fate restriction occurring in the caudal SHF.

## 6. The Atrioventricular Canal Develops into the AVN and Atrioventricular Ring Bundles

The possible developmental origin of the AVN, AVRBs, AVB and BBs has been inferred from the spatio-temporal expression patterns of key markers during heart morphogenesis. In the formed heart, GlN2 is detected in the AVN, AVB and BBs. Earlier in development, GlN2 is expressed in the interventricular ring (IVR), which includes the crest of the forming IVS and the right-sided hemisphere of the AVC. From the crest, GlN2^+^ cardiomyocytes extend at both sides into the trabecular ventricular walls. Together, this pattern resembles the probable precursor framework of the AVN, AVB and BBs [57]. Almost the same expression pattern was observed for the T-box transcription factor *Tbx3*, with the exception that *Tbx3* is expressed in the entire AVC (and SAN) [58]. In addition, a transgenic mouse line carrying an enhancer of chicken *Gata6* (*cGata6*) coupled to *LacZ* showed activity in the AVN and AVB cardiomyocytes at E14.5. The *cGata6* enhancer, which was also active in the AVC at E9.5, became active around E7.5 in the posterior region of the cardiac crescent [59]. Altogether, this suggested that the AVN progenitors originate from the AVC and can be traced back to the posterior part of the cardiac crescent.

Specification of the AVC involves local repression of the WM gene program by Tbx3 and the closely related T-box transcription factor Tbx2 [58,60,61]. Within the AVC, these factors are functionally redundant in maintaining the AVC myocardial phenotype and the lack of three or four functional alleles of *Tbx2*/*Tbx3* abrogates myocardial patterning of the AVC [61]. *Tbx2* expression is observed within the limbs of the cardiac crescent in mouse development [60]. *Tbx2*-driven Cre lineage analysis revealed that the AVN, AVRBs and base of the LV derive from the *Tbx2*^+^ population. However, the AVB, BBs and PVCS do not. This suggests that the mature AVB and PVCS, although forming a myocardial continuity with the AVN, segregates before the onset of *Tbx2* expression in the cardiac progenitors. The early segregation of the AVN and AVB has also been shown by a retrospective clone analysis using *nLaacZ* [62]. Furthermore, analysis of the *Tbx2* lineage at E8.5 shows only labeled cells in the *Tbx2*^+^ IFT. One day later, the *Tbx2*^+^ AVC cardiomyocytes are labeled with no labeled cells in the LV and newly formed atria [60]. This suggests that the *Tbx2*^+^ cardiogenic mesoderm first forms the IFT, which subsequently becomes AVC and finally the AVN and AVRBs. Interestingly, *Tbx2* expression in the cardiac crescent coincidences with *cGata6* enhancer activity [59,60]. Indeed, *cGata6*-Cre lineage analysis showed labeling of the AVN and AVB. In addition, the *cGata6* lineage contributes to the LV where the *cGata6* enhancer itself is not active, thus resembling the Tbx2^+^ lineage contribution to the LV. A limitation in both the *Tbx2*^+^ and *cGata6*^+^ lineage tracing experiments is that the AVN maintains expression of these markers at the moment of analysis, precluding the conclusion that the *Cre*^+^ cells earlier in development are the AVN progenitors [59,60]. However, the E8.5 and E9.5 *Tbx2*-Cre fate maps showed labeled IFT and AVC cardiomyocytes and therefore the progeny of these cells are labeled in the fate map analysis of the mature heart [60]. Furthermore, Tbx2 is required to maintain the AVC myocardial phenotype and therefore is likely to be involved in the specification of the AVC/AVN lineage [61]. In conclusion, the AVC originates from the posterior part of the cardiac crescent and gives rise to the AVN and AVRBs.

The *Tbx2* and *cGata6* enhancer lineage analyses did not exclude the possibility that other cell populations contribute to the mature AVN. Additional Cre-based genetic lineage experiments have been performed and exclude contributions from particular lineages to the AVN. *Mef2c-AHF-Cre* is expressed in the anterior part of the SHF at E8.5. Lineage analysis revealed that the *Cre*^+^ mesodermal cells give rise to the RV, OFT and IVS, including the AVB [63]. However, the *Mef2c-AHF-Cre* lineage did not contribute to the AVN at any developmental stage [60]. In fact, a sharp boundary was found between the *Tbx2*^+^-derived AVC/AVN and the *Mef2c-AHF-Cre*^+^-derived *Gja5*^+^ AVB, including the *Gja5*^+^ lower nodal cells. In the same study, a *Tbx18* lineage analysis was performed. *Tbx18* is expressed in the epicardium and SV. The *Tbx18-Cre*-mediated lineage analysis excluded a contribution of these *Tbx18*^+^ tissues to the AVN cardiomyocytes. However, the *Tbx18*^+^ epicardium does contribute to the connective tissue surrounding the AVN cardiomyocytes [64]. A *Wnt1*^+^ neural crest lineage analysis (*Wnt1-Cre*) suggested that the neural crest contributes cells to the VCS [65]. A more careful examination of the genetic fate map revealed that *Wnt1*^+^ cells do not contribute to the *Gja5*^+^ cardiomyocytes of the VCS. Within the *Gja5*^−^ *Tbx3*^+^ AVN cardiomyocytes, the majority of the neural crest cell descendants do not express *Tbx3*, suggesting that neural crest cells do not contribute to the AVN cardiomyocytes [62]. Recently, by *in ovo* dye labeling, SV cells were shown to contribute to the posterior region of the AVC. The initially physically separated SV and AVC structures fuse and form a continuity later in development, and part of the SV becomes incorporated into the posterior AVC [66]. This contradicts the *Tbx18* lineage analysis, which indicated that the SV cardiomyocytes did not contribute to the AVN cardiomyocytes [48,64]. An incomplete *Tbx18*^+^ fate map could explain this discrepancy. On the other hand, in the dye labeling experiment, the AVC was not identified using genetic markers. Hence, it is possible that not the AVC became dye labeled, but cardiomyocytes with an atrial identity [66]. In conclusion, the above-mentioned cell populations probably do not contribute to the AVN, and it is likely that the AVN forms only from the *Tbx2^+^* and *cGata6*^+^ posteriorly located cardiogenic mesodermal population that first forms the AVC and subsequently the AVN and AVRBs.

## 7. The Ventricular Conduction System Originates from the Embryonic Trabeculae and Interventricular Ring

The mature VCS has an asymmetrical morphology left and right of the IVS and specifically expresses *Gja5*, encoding Cx40, in contrast to the other ventricular cardiomyocytes and to the AVN. Cx40 plays a role in fast propagation of the electrical impulse through the VCS. The LBB consists of several branches running downwards. In contrast, the RBB consists of only one branch. The PVCS also differs between the LV and RV. In the LV, the PVCS is present on the IVS, while in the RV it extends towards the free wall of the RV [67].

The patterns of GlN2, *Tbx3* and enhancer of *cGata6* provided a good indication of the origin of the AVB and BBs, but not the PVCS [57,58,59]. A possible origin of the PVCS came from studying the spatio-temporal expression patterns of* Gja5* and *Nppa* [68]. In mouse (E9-9.5) and chicken chamber-forming hearts, *Gja5* is expressed transmurally in the LV wall, and to a limited extent in the forming RV. *Gja5* remains to be expressed in the ventricular trabeculae and becomes gradually downregulated in the forming compact myocardium from approximately E12-13 onwards. This spatio-temporal pattern indicated that the formed PVCS and the ventricular compact wall both originate from the *Gja5*^+^ (and *Nppa*^+^) embryonic ventricular wall. Furthermore, it suggested that the PVCS forms from trabecular myocardium by maintaining its phenotype, whereas the compact myocardium diverges [68]. The *Tbx3*^+^ AVB and proximal BBs initially do not express *Gja5*, but gradually induce expression after E13-14 of mouse development. Altogether, gene expression patterns in the early embryonic ventricle suggest that the AVB and proximal BBs originate from the GlN2^+^ and *Tbx3^+^* and *Gja5^−^* IVR, while the distal BBs and the PVCS originate from the *Gja5*^+^ and GlN2*^−^* and *Tbx3*^−^ trabeculae.

The first VCS lineage study showed that the PVCS and ventricular WM cardiomyocytes share a common progenitor pool [69], consistent with the suggested origin based on the spatio-temporal expression pattern of *Gja5*. Avian RV cardiomyocytes were labeled by microinjection of a reporter-carrying retrovirus that randomly integrated into the host genome. Clone analysis showed that PVCS cardiomyocytes are closely related to surrounding WM cardiomyocytes. The AVB was not labeled in this study [69]. The same group also addressed the origin of the AVB and BBs. The same approach was used and extended by injecting high titers of reporter-carrying adenoviruses into the pericardial cavity. Like the PVCS, the AVB and BBs originate from cardiomyocytes that contribute to both conductive cells and WM cardiomyocytes. Furthermore, they showed that VCS conductive cells share closer lineage relationships with surrounding WM cardiomyocytes than with more distant VCS conductive cells [19].

Retrospective clonal analysis (using transgene *nLaacZ*) and genetic inducible fate mapping (using *Gja5-CreER^T2^-IRESmRFP* and *R26R* reporter) experiments confirmed that the VCS and ventricular WM originate from a common progenitor pool [37]. In addition, a progressive restriction towards the VCS was observed followed by a limited outgrowth. Double transgenic embryos were generated containing *nLaacZ*, to determine clonal relationships between cells, and *Gja5*^+/GFP^, to discriminate the VCS. The mode of development of the VCS was determined from the clone cluster size and cluster composition (containing only *Gja5*^+^ conductive cells, only *Gja5*^−^ WM cardiomyocytes, or both). The analysis indicated that the VCS developed by specification to a conductive phenotype (*Gja5*^+^) followed by a limited outgrowth of several cell divisions. In addition, it was shown that the VCS develops by a progressive restriction. *Gja5*^+^ cardiomyocytes labeled at early developmental stages were observed to contribute to both *Gja5*^+^ VCS cells and *Gja5*^−^ WM cells. Labeling at later stages progressively resulted in larger contributions to *Gja5*^+^ VCS cells and reduced contributions to *Gja5*^−^ WM cells. Complete restriction of VCS progenitors to the VCS fate was established around E16.5 in mouse development [37].

It is still unclear from which population within the cardiogenic mesoderm the VCS originates. The *Mef2c-AHF-Cre* lineage tracing labels the RV, OFT and IVS, including the AVB. However, this lineage tracing experiment does not allow for tracing these contributions back to the *Mef2c-AHF*^+^ mesodermal cells because *Cre* remains expressed in the RV, IVS and AVB at the moment of fate analysis [63,64]. One study reporting a *Mesp1*^+^ fate map concluded that a fraction of the AVB and BBs in the IVS originate from a *Mesp1*^−^ mesodermal population [70]. This contradicts previous work showing that the myocardium of the entire E9.5 embryonic heart originates from *Mesp1*^+^ progenitor cells [71]. A possible explanation for this observation is incomplete labeling of the *Mesp1*^+^ population, as a result of low or brief *Mesp1* expression in certain cells, resulting in an incomplete fate map. As mentioned in the AVN section, a neural crest contribution to cardiomyocytes in the IVS is unlikely too [62,65].

## 8. Conclusions

Studies on mouse and chicken embryos using a variety of lineage tracing techniques have identified the locations of the progenitor cells of the CCS from the cardiogenic mesoderm to the formed heart. The findings clearly show that there are multiple CCS lineages. The progenitors of the SV/SAN cardiomyocytes segregate before they differentiate into cardiomyocytes in the most caudal part of the SHF and do not contribute to other cardiomyocyte populations. The posterior part of the cardiac crescent (a FHF and SHF-derived population) first form the IFT of the primary heart tube and then the AVC that in its turn gives rise to the AVN, AVRBs and part of the LV. The progenitors of the AVB and proximal BBs are added to the arterial pole of the primary heart tube that later forms the IVR/IVS and their origin is intimately related to the ventricular WM cardiomyocytes. The PVCS in the LV is a derivative of the first cardiogenic mesodermal cells (FHF) differentiating into cardiomyocytes that initially form the primary heart tube. The PVCS in the RV forms within the RV compartment and originates from the SHF. The SV/SAN, AVN/AVRBs, AVB and proximal BBs, PVCS in LV and PVCS in RV originate from separate lineages that segregate early in embryonic development due to cell fate decisions while remaining connected to each other. These early lineage segregations in the progenitor populations result in sharp boundaries between the CCS component they will form (Figure 1).
